# Molecular docking studies of quercetin and its analogues against human inducible nitric oxide synthase

**DOI:** 10.1186/2193-1801-1-69

**Published:** 2012-12-17

**Authors:** Salam Pradeep Singh, Bolin Kumar Konwar

**Affiliations:** Department of Molecular Biology and Biotechnology, Tezpur University, Tezpur, 784028 Assam India

**Keywords:** Inducible iNOS, Molecular docking, Molecular modification, Cancer, Analogues

## Abstract

Nitric oxide synthases (NOS) catalyze to produce nitric oxide (NO) from L-arginine. The isoform of NOS i.e. inducible nitric oxide synthases (iNOS) expression is observed in various human malignant tumors such as breast, lung, prostate and bladder, colorectal cancer, and malignant melanoma. Also an increased level of iNOS expression and activity has been found in the tumor cells of gynecological malignancies, stroma of breast cancer and tumor cells of head and neck cancer. Because of its importance in causing tumors and cancer, iNOS enzyme has become a new target in finding novel inhibitors as anti cancer agents. The present work focuses on the molecular docking analysis of quercetin and its analogues against iNOS enzyme. Earlier there are reports of quercetin inhibiting iNOS enzyme in certain experiments as anti cancer agent. But the clinical use of quercetin is limited by its low oral bioavailability and therefore needed its molecular modification to improve its pharmacological properties. In the present study ten analogues of quercetin were found to be docked at the active site cavity with favorable ligand-protein molecular interaction and interestingly from the ADME-Toxicity analysis these analogues have enhanced pharmacological properties than quercetin.

## Background

Nitric oxide synthases (NOS) (EC 1.14.13.39) are a family of enzymes that catalyze producing nitric oxide (NO) from L-arginine. It is an important cellular signaling molecule, having a role in various cellular processes. The free radical (NO) is an important effector molecule in the nervous, immune and cardio vascular systems (Garthwaite and Boulton 1995; MacMicking et al. 1997; Michel and Feron, 1997). Mammals contain three isoforms of NOS that produce NO and citrulline by catalyzing NADPH and O_2_ dependent oxidation of L-arginine (Griffith and Stuehr 1995; Marletta et al. 1997). Two isoforms of NOS are expressed in cells such as neurons (nNOS) and endothelium (eNOS) which are activated by Ca^+2^ dependent calmodulin (CaM) binding and the third inducible isoform (iNOS) is induced by cytokines which binds CaM independently (Ghosh et al., 1999). The iNOS isoform is a homodimer (Michal 1999) and the iNOS gene is located on chromosome 17 (Xu et al. 1994). iNOS exerts its functions independent of Ca^+2^ while calmodulin remains non-covalently bound to the iNOS complex and forms an essential subunit of the isoform (Knowles and Moncada 1994, Cho et al. 1992). Regulating NO production via iNOS necessarily occurs during transcription and translation, for once active, iNOS synthesizes large amounts of NO until substrate depletion (Hickey et al. 2001). The role of NO affects the expression and activity of oncogenes, which are vital to the cell cycle and apoptosis (Forrester et al. 1996; Messmer et al. 1994; Sandau et al. 1997). Forrester et al. observed an up regulation of the tumor suppressor gene p53 after the exposure of cells to NO donors which might be a reaction due to NO mediated DNA damage (Forrester et al. 1996). Also, the p53 gene is an important inhibitor for iNOS expression as it regulates NO production by a negative feedback loop mechanism. The non-mutant p53 protein (wild-type form) binds to a site on the iNOS gene, preventing its transcription (Brennan and Moncada 2002). Thus, suggesting the wild-type p53 is vital for the control of NO mediated genotoxicity (Forrester et al., 1996). Certain experiments with mutant p53 animal tumors have found out there is an increase in NOS activity in such cancers which grew faster with greater angiogenic potential. Thus, promoting cancer progression by providing a selective growth advantage to tumor cells (Ambs et al. 1998a). NO could also be shown to activate p53 resulting in anti-carcinogenic effects, mutagenic and increase cancer risk (Goodman et al. 2004, Rao 2004). The multifactorial process involved in carcinogenesis requires mutations in somatic cells and subsequent alterations of morphology and growth pattern, eventually resulting in transformation, local invasion, and metastasis (Lirk et al. 2002). The expression of iNOS can be observed in a various human malignant tumors such as breast (Vakkala et al. 2000), lung (Marrogi et al. 2000), prostate (Aaltoma et al. 2001; Aaltomaa et al. 2000; Uotila et al., 2001) and bladder (Swana et al. 1999; Hayashi et al. 2001), colorectal cancer (Kojima et al. 1999), and malignant melanoma (Massi et al. 2001). However, there are many conflicting reports that increased levels of iNOS are not a ubiquitous finding in human cancer and its expression depends on the histological type or grade of the tumor and the tumor stage (Crowell et al. 2003; Kinaci et al. 2012). Various studies have also found out the expression and the activity of iNOS in human cancer (Weiming et al. 2002; James et al. 2003). An increased level of iNOS expression and activity has been found in the tumor cells of gynecological malignancies, (Thomsen et al., 1994) in the stroma of breast cancer, (Thomsen et al. 1995) and in the tumor cells of head and neck cancer (Gallo et al. 1998; Franchi et al. 2002). Several studies have reported an increase of iNOS expression in tumor tissue when compared with normal mucosa (Ambs et al. 1998b; Ambs et al. 1999; Ropponen et al. 2000; Yagihashi et al. 2000; Kojima et al. 1999; Hao et al. 2001).

The present work aims on molecular docking analysis of iNOS enzyme against a class of flavonoid (quercetin and its analogues) which is present in fruits, vegetables, leaves and grains and is reported to have effective anti-cancer property. Scientists have long considered quercetin and flavonoids present in fruits, vegetables, leaves and grains important in cancer prevention. There are also reports of lower risk of cancer in people who eat more fruits and vegetables. (Verschoyle et al. 2007; Rietjens et al. 2005; van der Woude et al. 2005; Chen et al. 2001). Interestingly, quercetin inhibiting against iNOS as anti cancer agents has been reported by García-Mediavilla et al. and Raso et al. (García-Mediavilla et al. 2007; Raso et al. 2001). But the clinical use of quercetin is limited by its low oral bioavailability (Peng et al. 2008) and therefore compels its molecular modification to enhance its pharmacological properties. In the present study the best docking hit analogues were undergo ADME–Toxicity prediction (absorption, distribution, metabolism, and toxicity) to evaluate its pharmacological properties to be an orally active compound. Here in the present work, we are reporting for the first time the analogues of quercetin as iNOS inhibitors with enhanced pharmacological properties.

## Results and discussion

### Molecular docking analysis

Quercetin (3,3’,4’,5,7-pentahydroxylflavone) is a plant derived flavonoid which is present in the plant kingdom as a secondary metabolite. It is the most well defined group of polyphenolic compounds (Murakami et al., 2008). The flavonoids contain a basic skeleton of diphenylpropane (C6–C3–C6). Quercetin is commonly found as O-glycosides with one of its hydroxyl group is substituted by sugars of various type. In this report, we have highlighted molecular docking studies on the inhibition of iNOS by quercetin and its analogues. Molecular docking was carried out using Molegro Virtual Docker, MVD 5.0 (Molegro 2011). The top poses were found to be lying deep into the binding cavity of iNOS enzyme showing all the major interaction and a favourable interaction energy than quercetin ranging from −130.62 to −150.44 compared with −97.17of quercetin. The top docking hits were bound within the active site cavity consisting of the protoporphyrin IX containing Fe (HEM) revealing molecular interaction with the active site residues and HEM. The analogues docked at the binding cavity have a rerank score ranging from −104.75 (CID5281604) to −65.79 (quercetin) as shown in Table 1. The rerank score is a linear combination of E-inter (steric, Van der Waals, hydrogen bonding, electrostatic) between the ligand and the protein, and E-intra (torsion, sp2-sp2, hydrogen bonding, Van der Waals, electrostatic) of the ligand weighted by pre-defined coefficients (Thomsen and Christensen 2006). Also, the top three docking hits have a MolDock score of −129.14 for CID5281604, −122.90 for CID5315126, −133.99 for CID9818879 and −77.29 for quercetin.

**Table 1:** **Docking score of the top docking hits and quercetin**

SN	Ligand	MolDock^a^	Rerank^b^	Interaction^c^	Internal^d^	HBond^e^	LE1^f^	LE3^g^
1	5281604	−129.14	−104.75	−148.27	19.14	−11.81	−5.61	−4.55
2	5315126	−122.90	−102.63	−146.11	23.21	−15.38	−4.55	−3.80
3	9818879	−133.99	−95.04	−150.44	16.46	−9.33	−5.58	−3.96
4	5481966	−122.87	−93.67	−141.73	18.86	−2.43	−4.55	−3.47
5	5282154	−116.71	−93.58	−135.98	19.27	−14.02	−4.86	−3.90
6	13964550	−113.94	−93.40	−130.62	16.68	−4.56	−5.18	−4.25
7	5281691	−124.63	−92.63	−144.57	19.94	−7.95	−5.42	−4.03
8	11834044	−116.92	−91.50	−140.67	23.75	−13.46	−5.08	−3.98
9	6477685	−130.50	−91.09	−144.13	13.63	−4.18	−5.67	−3.96
10	Quercetin	−77.29	−65.79	−97.17	19.88	−8.42	−3.51	−2.99

a - Moldock score is derived from the PLP scoring functions with a new hydrogen bonding term and new charge schemes. (Thomsen and Christensen 2006).

b - The rerank score is a linear combination of E-inter (steric, Van der Waals, hydrogen bonding, electrostatic) between the ligand and the protein, and E-intra. (torsion, sp2-sp2, hydrogen bonding, Van der Waals, electrostatic) of the ligand weighted by pre-defined coefficients. (Thomsen and Christensen 2006).

c - The total interaction energy between the pose and the protein (kJ mol^−1^).

d - The internal energy of the pose.

e - Hydrogen bonding energy (kJ mol^−1^).

f - Ligand Efficiency 1: MolDock Score divided by Heavy Atoms count.

g - Ligand Efficiency 3: Rerank Score divided by Heavy Atoms count.

The MolDock scoring function (MolDock Score) is derived from the PLP scoring functions originally proposed by Gehlhaar et al. (Gehlhaar et al. 1995, Gehlhaar et al. 1998) and later extended by Yang et al. (Yang and Chen 2004). The MolDock scoring function further improves these scoring functions with a new hydrogen bonding term and new charge schemes. The docking scoring function, E_score_, is defined by the following energy terms:

Where,

E_*inter*_ is the ligand-protein interaction energy

E_*intra*_ is the internal energy of the ligand

Also the hydrogen bonding energy which describes the binding affinity for the docked compounds ranges from −15.38 kJ mol^-1^ for CID5315126 to −2.43 for CID5481966 while quercetin have a hydrogen bonding energy of −8.42 kJ mol^-1^.

The ligand-protein interaction analysis for the top ten docking hits was calculated using MVD ligand energy inspector. The ligand–protein interaction including the residues present, their interaction distances and interaction energy and the interacting atoms of the protein and the ligand is shown in Table 2. The molecular docking simulation revealed that the top docking poses were found to be docked into the binding cavity displaying both bonded and non bonded interaction.

**Table 2:** **Molecular interaction analysis of the top three docking hits and quercetin**

SN	Compound ID	Interacting Atom ID and Name (Ligand)	Interacting Atom Name (Protein/Cofactor)	Interaction Energy (kJ mol^−1^)	InteractionDist. (Å)
1	**CID5281604**	5(O)	O(Phe369)	−2.43	3.04
		5(O)	N(Val352)	−1.71	3.26
		4(O)	OD2(Asp382)	−2.5	2.97
		4(O)	NE1(Trp346)	−0.18	3.54
		4(O)	OH(Tyr347)	−2.5	3.0
		4(O)	OH(Tyr373)	−2.5	2.77
		8(O)	N(HEM)	−2.5	3.10
		8(O)	N(HEM)	−2.5	2.87
2	**CID5315126**	3(O)	NE1(Trp346)	−0.02	3.59
		3(O)	OH(Tyr347)	−2.5	3.06
		3(O)	OH(Tyr373)	−2.11	2.55
		3(O)	OD2(Asp282)	−2.5	3.07
		1(O)	OD1(Asp382)	−2.5	2.65
		1(O)	NH2(Arg388)	−1.1	3.26
		1(O)	NH1(Arg388)	−1.78	3.10
		6(O)	O(Pro350)	−1.98	2.54
		6(O)	N(Gly371)	−0.88	2.77
		2(O)	O(HEM)	−2.5	2.60
		4(O)	N(HEM)	−1.03	3.39
3	**CID9818879**	4(O)	OD1(Asp382)	−2.0	3.07
		4(O)	OD2(Asp382)	−1.7	3.09
		5(O)	OD1(Asp382)	−2.5	3.10
		5(O)	OH(Tyr347)	−1.8	3.25
		3(O)	O(Pro350)	−1.4	3.31
		6(O)	O(HEM)	−2.5	3.10
		6(O)	O(HEM)	−2.5	2.77
4	**Quercetin**	6(O)	OD1(Asp382)	−2.5	2.60
		6(O)	NH1(Arg388)	−2.26	3.08
		5(O)	NE2(Gln263)	−0.34	2.35
		4(O)	O(Pro350)	−2.5	2.75
		4(O)	N(Gly371)	−0.82	2.66
		1(O)	O(HEM)	−2.5	3.10
		1(O)	O(HEM)	−2.5	2.68
		2(O)	N(HEM)	−0.4	3.52

HEM - Protoporphyrin IX containing Fe.

**Figure 1:**
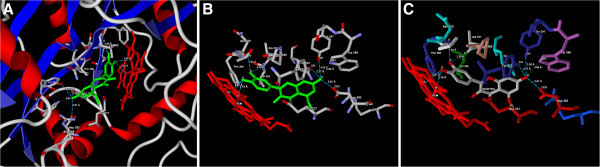
**(A) Predicted bonded interactions (green dashed lines) between CID5281604 (green) and Trp346, Tyr347, Val352, Phe369, Tyr373, Asp382 residues and HEM molecule of iNOS (B) binding mode of CID5281604 (green) to iNOS active site region (C) Binding mode representing the ligand based on atom type and the protein based on amino acid residue type colouring.**

**Figure 2:**
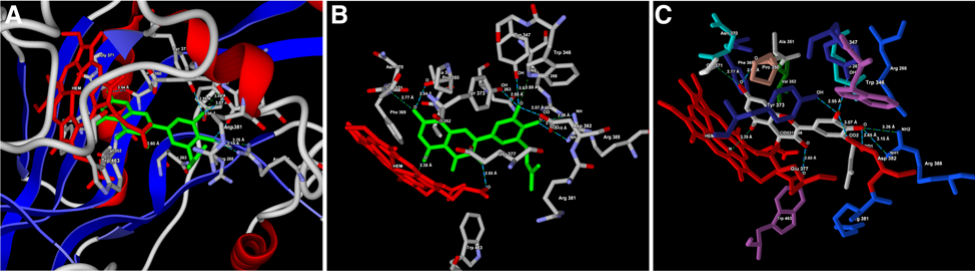
**(A) Predicted bonded interactions (green dashed lines) between CID5315126 (green) and Asp282, Trp346, Tyr347,Pro350, Gly371, Tyr373, Asp382, Arg388 residues and HEM molecule of iNOS (B) Binding mode of CID5315126 (green) to iNOS active site region (C) Binding mode representing the ligand based on atom type and the protein based on amino acid residue type colouring.**

**Figure 3:**
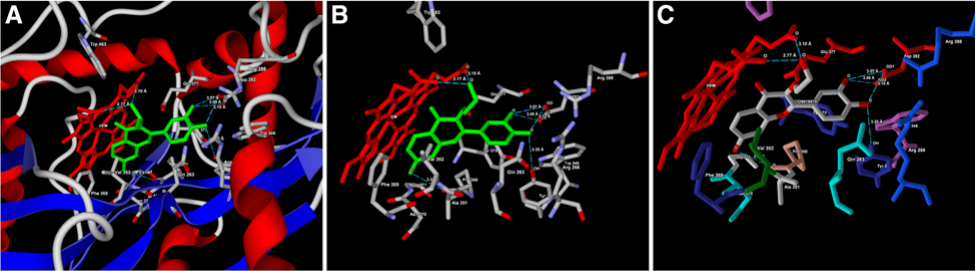
**(A) Predicted bonded interactions (green dashed lines) between CID9818879 (green) and Asp382, Tyr347, Pro350 residues and HEM molecule of iNOS (B) Binding mode of CID9818879 (green) to iNOS active site region (C) Binding mode representing the ligand based on atom type and the protein based on amino acid residue type colouring.**

**Figure 4:**
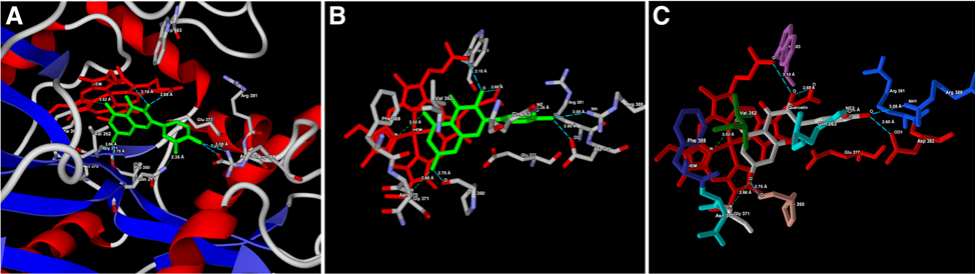
**(A) Predicted bonded interactions (green dashed lines) between quercetin (green) and Gln263, Pro350, Gly371 Asp382, Arg388 residue and HEM molecule of iNOS (B) Binding mode of quercetin (green) to iNOS active site region (C) Binding mode representing the ligand based on atom type and the protein based on amino acid residue type colouring.**

The top three docking hits showed common molecular interaction with Asp382, Tyr347 and HEM molecule. The snapshots of ligand-protein interaction and the binding mode for the top three docking hits (CID44610309, CID44259709, CID13964550) and quercetin is shown in Figure 1A,B,C, Figure 2A,B,C, Figure 3A,B,C and Figure 4A,B,C.

The Lipinski rule of five parameters for the top docking hits and quercetin is shown in Table 3. Lipinski rule of five is a rule to evaluate drug likeness to determine if a chemical compound has a certain pharmacological or biological activity to make it an orally active drug in humans (Lipinski 2008; Lipinski et al. 1997). It is observed from Table 3, the hydrogen bond acceptor (HBA) of quercetin is very low (only one HBA) compared to HBA of the top docking hits (6–8 HBA). The high number of HBA of the analogues could be an important factor and hence the analogues showed better binding affinity and molecular interaction with iNOS enzyme compared to quercetin. Additionally, the top docking hits have lower topological surface area (TPSA) values than quercetin suggesting that these compounds might have better oral bioavailability compared to quercetin (the oral bioavailability is inversely proportional to topological polar surface area) (Freitas 2006).

**Table 3:** **Lipinski rule of five filter including TPSA for the top poses**

SN	Compound ID	HBA	HBD	Mol. Wt.	XLog P3	Rot B	TPSA
1	5281604	7	4	316.26	1.3	2	116
2	5315126	7	5	370.35	3.5	3	127
3	9818879	7	4	330.29	1.6	4	116
4	5481966	7	5	370.35	4.1	3	127
5	5282154	8	5	332.26	1.5	2	137
6	13964550	6	3	300.26	1.2	2	96.2
7	5281691	7	4	316.26	1.9	2	116
8	11834044	7	4	316.26	2.5	2	116
9	6477685	6	4	312.27	2.0	2	107
10	Quercetin	1	5	302.23	1.5	1	127

### ADME-toxicity analysis

The QuikProp (Schrödinger 2012) prediction for the top docking hits and quercetin is shown in Table 4. From Table 4, it is revealed that the top docking hits have MDCK cell permeability (QPPMDCK) in the acceptable range except for quercetin and CID9818879, the docked compounds are also in permissible range for IC_50_ value for blockage of HERG K^+^ channels (QPlogHERG), Caco-2 cell permeability (QPPCaco) and brain/blood partition coefficient (QPlogBB). More interestingly, the top docking hits showed higher human oral absorption (PercentHuman-OralAbsorption) ranging from 58.62% (CID5282154) to 69.077% (CID5481966) compared with 53.424% of quercetin.

**Table 4:** **ADME and pharmacological parameters prediction for the top docking hits using QikProp**

SN	CID^a^	QPPMDCK^b^	QPlogHERG^c^	QPPCaco^d^	Rule of 5^e^	QPlogBB^f^	PercentHuman- OralAbsorption^g^	QPlogS^h^
1	5281604	46.45	−5.78	112.076	0	−1.997	62.147	−2.657
2	5315126	39.535	−5.519	96.549	0	−2.035	67.901	−3.821
3	9818879	15.081	−5.291	39.584	0	−2.253	59.194	−2.99
4	5481966	44.662	−5.886	108.08	0	−2.08	69.077	−4.04
5	5282154	33.312	−4.486	82.401	0	−1.817	58.62	−1.669
6	13964550	41.929	−6.124	101.947	0	−2.086	64.314	−3.349
7	5281691	63.477	−4.866	149.622	0	−1.572	66.37	−2.045
8	11834044	43.054	−4.535	104.474	0	−1.543	61.02	−1.685
9	6477685	45.568	−4.337	110.106	0	−1.605	64.219	−1.197
10	Quercetin	20.486	−4.032	52.551	0	−1.754	53.424	−1.169

^a^Compound I.D’s from NCBI PubChem database.

^b^Predicted apparent MDCK cell permeability in nm/s (acceptable range: <25 is poor, >500 is great).

^c^Predicted IC_50_ value for blockage of HERG K^+^ channels (concern below −7).

^d^Predicted Caco-2 cell permeability in nm/s (acceptable range: <25 is poor, <500 is great).

^e^Number of violations of Lipinski’s rule of five (Lipinski et al. 1997; Bracket should be closed.

^f^Predicted brain/blood partition coefficient (Concern value is–3.0 to – 1.2).

^g^Predicted human oral absorption on 0 to 100% scale (acceptable range: <25% is poor, >80% is high).

^h^Predicted aqueous solubility, (Concern value is −6.5 to – 0.5).

Also, the top docking hits used in the present study does not violate Lipinski rule of five parameters. Lipinski rule of five is a rule to evaluate drug likeness to determine if a chemical compound has a certain pharmacological or biological activity to make it an orally active drug in human (Lipinski 2008; Lipinski et al. 1997). However, the rule does not predict whether a compound is pharmacologically active.

**Figure 5:**
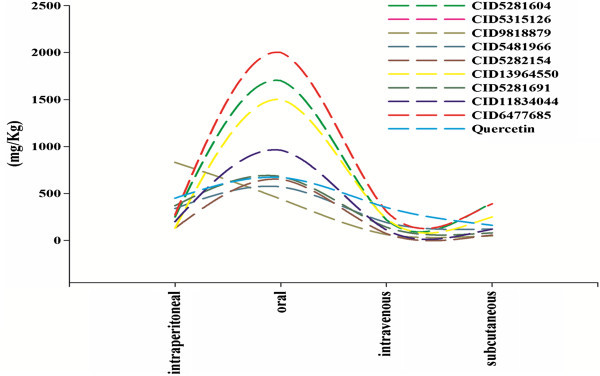
**Comparative analysis on LD**_**50 mouse**_**(intraperitoneal, oral, intravenous, subcutaneous) for Compound ID (5281604, 5315126, 9818879, 5481966, 5282154, 13964550, 5281691, 11834044, 6477685 and quercetin).**

**Figure 6:**
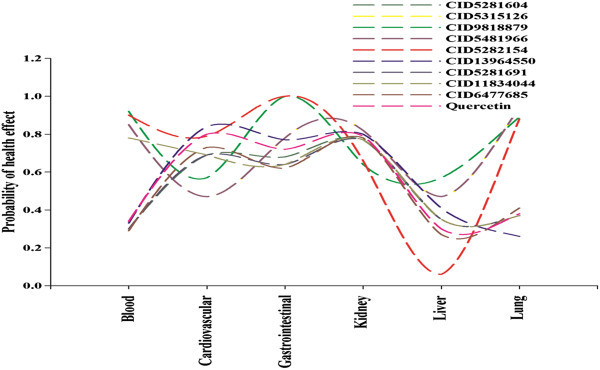
**Comparative analysis on probability of health effect on blood, cardiovascular system, gastrointestinal system, kidney, liver and lung for Compound ID (5281604, 5315126, 9818879, 5481966, 5282154, 13964550, 5281691, 11834044, 6477685 and quercetin).**

Again from the LD_50 mouse_ and probability of health effects predictions for the top docking hits and quercetin using ACD/ I-Lab 2.0 (Advanced Chemistry Development, Inc 1994) revealed the top docking hits have lower LD_50_ and lesser chance of health effects (shown in Table 5). The comparative analysis on the LD_50_ oral revealed CID11834044, CID13964550, CID5281604 and CID 6477685 have higher LD_50_ oral compared to quercetin (shown in Figure 5). Additionally, the comparative analysis on probability of health effects showed the top docking hits have more or less similar behaviour of health effects with quercetin except for CID5282154 and CID9818879 which showed chances of health effect on gastrointestinal system and lung (shown in Figure 6). In short, the top docked compounds could be lead molecule or a potential anti-cancer compound with enhanced pharmacological properties as compared to quercetin.

**Table 5:** **LD**_**50**_**and probability of health effects prediction for the docking hits and quercetin using ACD/ I-Lab 2.0**

ADME-Tox parameters	5281604	5315126	9818879	5481966	5282154	13964550	5281691	11834044	6477685	Quercetin
LD^a^_50 mouse_ (mg kg^−1^, intraperitoneal)	250	340	830	340	130	130	370	200	270	450
LD^a^_50 mouse_ (mg kg^−1^, oral)	1700	570	440	570	650	1500	680	960	2000	670
LD^a^_50 mouse_ (mg kg^−1^, intravenous)	220	190	62	190	71	230	140	110	310	350
LD^a^_50 mouse_ (mg kg^−1^, subcutaneous)	400	120	48	120	57	250	80	120	390	160
Prob. of blood effect^b^	0.3	0.85	0.92	0.85	0.9	0.33	0.3	0.78	0.29	0.34
Prob. of cardiovascular system effect^b^	0.69	0.47	0.57	0.47	0.79	0.84	0.69	0.69	0.73	0.8
Prob. of gastrointestinal system effect^b^	0.68	0.78	1	0.78	1	0.77	0.64	0.64	0.62	0.72
Prob. of kidney effect^b^	0.77	0.82	0.64	0.82	0.66	0.8	0.77	0.77	0.78	0.79
Prob. of liver effect^b^	0.35	0.47	0.57	0.47	0.06	0.41	0.35	0.35	0.27	0.3
Prob. of lung effect^b^	0.37	0.94	0.89	0.94	0.88	0.26	0.37	0.37	0.41	0.38

a- Estimates LD_50_ value in mg/kg after intraperitoneal, oral, intravenous and subcutaneous administration to mice.

b- Estimates probability of blood, gastrointestinal system, kidney, liver and lung effect at therapeutic dose range.

## Conclusions

The molecular docking studies with quercetin and its analogues into the binding cavity of iNOS inducible showed the analogues having more favourable interaction than quercetin with better rerank score, docking score, hydrogen bonding energy and ligand-protein interaction energy compared to quercetin. As earlier reported in literature, quercetin is known for having anti-cancer property and inhibiting the iNOS enzyme, the analogues docked at the binding cavity could have also possess some sort of anti-cancer property as it is 95% similar to quercetin retrived form the NCBI PubChem database. The docked compounds used in the present study do not violate the Lipinski rule of five parameters.

Also, from the ADME-Toxicity prediction using QikProp and ACD/ I-Lab 2.0 revealed the docked compounds are in the acceptable range of various pharmacological parameters and they have similar behaviour of health effects and LD_50_ compared to quercetin. Interestingly, the top dockings showed higher human oral absorption ranging from 58.62% (CID5282154) to 69.077% (CID5481966) compared with 53.424% of quercetin which is primary concern of this study as the clinical use of quercetin is limited by its low oral bioavailability.

Therefore we conclude that these compounds could be a potential lead molecule and supports for experimental testing against iNOS enzyme as anti cancer compounds.

## Methods

### Protein preparation

The three-dimensional crystal structure of human inducible nitric oxide synthase (PDB ID: 4NOS) was retrieved from the Protein Databank Bank (http://www.rcsb.org/). The coordinates of the dimeric crystallized iNOS is complexed with water molecules, iron protoporphyrin IX (heme), BH_4_, Zn^+2^ atom, ethylisothiourea and has a resolution of 2.25 Å. (Fischmann et al.^1999^). For molecular docking purpose, the dimeric molecule and iron protoporphyrin IX (heme) was loaded in the Molego Virtual Docker (MVD) and all the water molecules were removed.

### Chemical similarity search

The 2D structure of quercetin (CID5280343) was retrieved from the NCBI PubChem database (Bolton et al. 2008; Wang et al. 2010) and performed a chemical structure search of quercetin at the NCBI PubChem database to retrieve the related compound and analogues. The search parameters were set at 95% similarity subjected to Lipinski rule of five filters (Lipinski et al. 1997; Lipinski 2008) resulting with 85 compounds.

The retrieved compounds were converted to three-dimensional format using the ChemOffice 2010 (ChemOffice 2010: CambridgeSoft Corporation) for docking purposes. The energy of these compound were optimized using MM2 force field methods (Ulrich and Norman 1982) and save as sybyl mol2 file format using ChemOffice 2010.

### Computation

Potential ligand binding site for iNOS dimer (PDB ID: 4NOS) was predicted using MVD, having a volume of 678.91 Å^3^ and a surface area of 1245.44 Å^2^. The binding site was set inside a restriction sphere of radius 15 Å (X 0.28, Y 99.79, Z 8.70) using MVD.

The 85 analogues retrieved from the NCBI PubChem database were imported in the Molegro Virtual Docker (MVD). Bond flexibility of the compounds was set along and the side chain flexibility of the protein for the active site residues (Trp372, Glu377, Trp463, Phe476) was set with a tolerance of 1.10 and strength of 0.90 for docking simulations. RMSD threshold for multiple cluster poses was set at 2.00 Å. The docking algorithm was set at a maximum iteration of 1,500 with a simplex evolution size of 50 and a minimum of 10 runs were performed for each compound. The best pose of each compound was selected for the subsequent ligand–protein interaction energy analysis.

Molecular docking was carried out using Molegro Virtual Docker. MVD is based on a differential evolution algorithm; the solution of the algorithm considers the sum of the intermolecular interaction energy between the ligand and the protein and the intramolecular interaction energy of the ligand. The docking energy scoring function is based on the modified piecewise linear potential (PLP) with new hydrogen bonding and electrostatic terms included. Full description of the algorithm and its reliability compared to other common docking algorithm is described by Thomsen et al. (Thomsen and Christensen 2006).

### ADME-toxicity prediction

ADME-Toxicity for the top docking hits and quercetin was predicted using QikProp (Schrödinger 2012). QikProp predicts physically significant descriptors and pharmaceutically relevant properties of organic molecules, either individually or in batches. QikProp provides ranges for comparing a particular molecule’s properties with those of 95% of known drugs. In the present study QikProp properties and descriptors such as apparent MDCK cell permeability (QPPMDCK), IC_50_ value for blockage of HERG K^+^ (QPlogHERG), Caco-2 cell permeability (QPPCaco), Lipinski rule of five (Lipinski et al. 1997; Lipinski 2008), brain/blood partition coefficient (QPlogBB), human oral absorption on 0 to 100% scale (Percent HumanOralAbsorption), aqueous solubility (QPlogS) for the top docking hits and quercetin was predicted to obtain the ADME properties of the compounds.

Additionally LD_50 mouse_ and probability of health effects predictions for the top docking hits were calculated using ACD/ I-Lab 2.0 (Advanced Chemistry Development, Inc 1994) which is a web-based service that provides instant access to spectral and chemical databases, and predicts properties including physicochemical, ADME, toxicity characteristics. Also a comparative analysis were performed for LD_50 mouse_ (intraperitoneal, oral, intravenous, subcutaneous) and probability of health effect of blood, cardiovascular system, gastrointestinal system, kidney, liver and lung for the top docking hits.

## Abbreviations

NO: Nitric oxide
NOS: Nitric oxide synthases
iNOS: Inducible Nitric oxide synthase
eNOS: Endothelium Nitric oxide synthases
(nNOS): Neurons Nitric oxide synthase
CaM: Ca+2 –dependent calmodulin
MVD: Moegro Virtual Docker
ADME–Toxicity: Absorbtion distribution metabolism excretion and toxicity
NCBI: National Centre for Biotechnology Information
ACD: Advance Chemistry Development.
